# Unilateral Vulvar Pneumatocele (Pneumolabium) Diagnosed during Robotic Hysterectomy

**DOI:** 10.1155/2019/8106451

**Published:** 2019-12-10

**Authors:** Dani Zoorob, Mahala Spalsbury, Tiffany Slutz, Madeleine Oliver, Ibrahim Tsolakian, Jeffrey Judis

**Affiliations:** ^1^University of Toledo, Department of Obstetrics and Gynecology Academic Offices, 2142 N. Cove Blvd, Toledo, OH 43606, USA; ^2^ProMedica Health System, Sunforest Obstetrics and Gynecology, 5700 Monroe Street, Suite 303, Sylvania, OH 43560, USA

## Abstract

**Background:**

Persistent canal of Nuck may manifest in adulthood and be detected after increased abdominal pressure.

**Case:**

A 52-year -old female undergoing a robotic hysterectomy developed an acute left labial enlargement. The patient was discovered to have an 8 × 4 × 4 cm swelling in the left labium majus. Palpation revealed the mass to be air-filled and reducible by gentle compression with slow redevelopment upon release of the pressure while intra-abdominal insufflation was maintained.

**Conclusion:**

We present the first case of a labial pneumatocele during robotic hysterectomy. We theorize that the underlying cause to be a patent canal of Nuck that, once supplied with increasing abdominal peritoneal pressure, allowed air to travel into the labia majora through the inguinal canal. Simple reduction corrected the pneumatocele and no complications or recurrence was noted postop. This case suggests that pathologies of the canal of Nuck should be considered in the differential of an adult presenting with inguinal or genital swelling.

## 1. Introduction

The canal of Nuck is an evagination of parietal peritoneum that extends through the internal inguinal ring and travels anterior to the round ligament to the labium majus [1]. It is homologous to the male processus vaginalis and is normally obliterated within the first year of life. Incomplete obliteration of the Canal of Nuck, a rare finding in itself, most often presents as painless hydrocele or an inguinal hernia in adolescence [2]. The persistence of the canal of Nuck in adulthood has been attributed to pathologies similar to hydroceles and hernias including reproductive organs; however, the prevalence of such patency has not been well established [3].

In males, pneumoperitoneum may manifest as a scrotal pneumatocele whether it be related to intra-abdominal procedures or due to bowel perforation [4, 5]. Often times, a pneumoscrotum is the first clinical sign of a perforated bowel or an intra-abdominal pathology [5]. These patients present with painless, translucent scrotal swelling that is completely reducible. In these cases, the air travels through a patent processus vaginalis, producing the physical manifestation.

While both labial and scrotal pneumatoceles are uncommon, their presence in the context of a pneumoperitoneum may be a sign of abnormal pathology suggestive of hernias, surgical trauma, or potentially patent canal of Nuck.

## 2. Clinical Case

A 52-year -old premenopausal G6P3033 female presented with abnormal uterine bleeding that began in early 2018. Dilation and curettage (D&C) pathology showed endometrial hyperplasia without atypia. After failed medical management with Norethindrone, a robotic hysterectomy was planned.

The patient underwent general endotracheal anesthesia and was placed in dorsal lithotomy in Allen stirrups for the procedure. A 10-mm skin incision was made just above the umbilicus and open (Hassan) technique was used to enter the peritoneal cavity atraumatically. The 10 mm blunt balloon trocar was placed and CO_2_ was used to insufflate the peritoneal cavity to 15 mmHg. Two additional trocars were placed 10 cm bilaterally to the first and finally an assistance port was placed between the left lateral and umbilical trocars under direct visualization without complication. The intestines were mobilized in Trendelenburg before the DaVinci robot was then docked with targeting. Using fenestrated bipolar and monopolar scissors, the tubo-ovarian ligaments were cauterized and cut down over the round ligament, the bladder was dissected and the uterine arteries, cardinal ligaments, and uterosacral ligaments were cauterized. After colpotomy was completed, the uterus was delivered through the vagina. The fallopian tubes were then cauterized, cut, and dropped into the vagina for removal. Finally, the vaginal cuff was closed with 0 V lock suture. At this point, there was no evidence of bleeding or ureteral injury, and insufflation was not yet turned off.

Upon inspection of the vulva, a new onset isolated left labium enlargement was noted. The variation was significant (8 × 4 × 4 cm) compared to preoperative status and the patient's right labium. The mons pubis did not appear enlarged and neither did the skin in the groin area or left lower abdominal region at the level of the inguinal ligament. No bleeding was noted, and no skin discoloration was noted either to possibly suggest a hematoma. Patient vital signs remained stable. Palpation suggested the labium to be air-filled. Pressure was gently applied directly to the left labium, and the swelling was completely reduced. No exudate or blood was visualized while the robotic camera was inspecting left aspect of the pelvis and abdominal wall. Manual pressure was removed and within a few minutes, the labium was noted to slowly swell up again starting near the middle to lower third of the labium and then expanding to involve the whole labium majus. The area involved was the same as prior to the application of the manual compression. Repeat laparoscopic inspection of the left inguinal region, including the round ligament, using the camera placed infraumbilically did not suggest the presence of any identifiable abnormalities. At this time and since the procedure was complete, insufflation was stopped and the peritoneal cavity was slowly decompressed back to normal pressure. The labial enlargement was manually reduced by topical pressure application, and it did not recur upon release. The patient was transferred to recovery in good condition and managed conservatively with Foley catheter and ice packs and kept for observation. The next morning, the patient was doing well with appropriate tenderness at the abdominal incision sites. There was no tenderness noted at the left labium or evidence of recurrence of the swelling. She was discharged later that same day after passing the voiding trial.

## 3. Discussion

The patent canal of Nuck is a rare finding that may manifest as a painless mass in childhood or adolescence. Should the Canal of Nuck persist asymptomatically into adulthood, the prevalence may be significantly underappreciated. Hydrocele of the canal of Nuck has been previously documented in an adult presenting for surgical correction of painless swelling in the groin [6]. Presence of the canal of Nuck may be visualized by MRI [2] but diagnostic imaging is often not performed due to the relatively rare nature of the condition, leaving the prevalence of the canal of Nuck in the asymptomatic adult largely unknown. Due to the undisclosed nature of the canal's persistence, this case illustrates how a patent canal of Nuck may unexpectedly reveal itself following insufflation in laparoscopic abdominal surgery.

Vulvar pneumatocele, appearing first time during laparoscopic procedures has been mentioned in the literature, but no associated references were found, hence it is a rare incidence [7]. The pathophysiology of vulvar pneumatocele has been compared to that of a scrotal pneumatocele which can be caused by weakly adherent walls of the processus vaginalis, getting torn away due to air pressure [8]. This theory is in concordance with our observation, where the pneumatocele was formed during a laparoscopic procedure and resolved at the end of the surgery.

Although rare, laparoscopic surgery being directly implicated in swelling has been reported in a handful of cases [9]. One study reported unilateral labial swelling in three patients within 24 hours following laparoscopic surgery, which interfered with their ability to void. They were treated with ice packs applied to labia, placement of foley catheters for urinary drainage, and bedrest with resolution of symptoms in 1–3 days. One proposed cause of this vulvar edema was the possibility of incomplete removal of irrigation fluid that escaped intraoperatively or postoperatively through a low supra-symphyseal puncture site, and travelled through the subcutaneous tissue to the labia. Others have suggested postoperative labial swelling could be due to the formation of a fistulous tract from improper trochar placement. These theories are unlikely the cause of labial swelling in our case, as there was complete resolution of the labial swelling after discontinuation of abdominal insufflation and lack of postoperative edema. Our case highlights the possibility of a patent canal of Nuck in the etiology of unexpected labial swelling during or following laparoscopic surgeries.

## 4. Conclusion

We presented the case of a vulvar pneumatocele appearing for the first time during robotic surgery, and self-resolving at the end of the procedure. The patient had a smooth postoperative course and was discharged the day after the surgery, and the pneumatocele did not lead to complications. Vulvar pneumatocele, a self-resolving benign entity, caused by a patent canal of Nuck, should be on the differential in patients with inguinal or labial swelling especially following procedures with abdominal insufflation.

## Additional Points

*Teaching Points.* (1−3 lessons for clinical management). The differential of a patent canal of Nuck should be given consideration in a patient presenting with a labial or inguinal swelling. Acute onset of labial swelling that presents in the setting of increased abdominal pressure, as seen in laparoscopic or robotic surgical cases should raise suspicion for the possibility of a patent canal of Nuck. Relief of labial swelling upon return to normal intra-abdominal pressure and steady pressure over the area can help support this diagnosis.

## References

[1] Anderson C. C., Broadie T. A., Mackey J. E., Kopecky K. K. (1995). Hydrocele of the canal of nuck: ultrasound appearance. *The American Surgeon*.

[2] Manjunatha Y. C., Beeregowda Y. C., Bhaskaran A. (2012). Hydrocele of the canal of nuck: imaging findings. *Acta Radiologica Short Reports*.

[3] Tubbs R. S., Loukas M., Shoja M. M., Salter E. G., Oakes W. J. (2007). Indirect inguinal hernia of the urinary bladder through a persistent canal of nuck: case report. *Hernia*.

[4] Ratan S. K., Ratan J., Gupta D. K. (1999). Scrotal pneumatocele secondary to colonic perforation. *Journal of Urology*.

[5] Coppes M. J., Roukema J. A., Bax N. M. A. (1991). Scrotal pneumatocele: a rare phenomenon. *Journal of Pediatric Surgery*.

[6] Kim K. S., Choi J. H., Kim H. M. (2016). Hydrocele of the canal of nuck in a female adult. *Archives of Plastic Surgery*.

[7] Heller D. S., Wallach R. C. (2007). *Vulvar Disease: A Clinicopathological Approach*.

[8] Burman David (1954). Scrotal pneumatocele. *British Medical Journal*.

[9] Pados G., Vavilis D., Pantazis K., Agorastos T., Bontis J. N. (2005). Unilateral vulvar edema after operative laparoscopy: a case report and literature review. *Fertility and Sterility*.

